# Revolutionizing Pulpectomy: An Observational Overview of Multigenerational Kedo Rotary File Systems in Primary Molars

**DOI:** 10.7759/cureus.65147

**Published:** 2024-07-22

**Authors:** Balaji Suresh, Ganesh Jeevanandan, Vignesh Ravindran

**Affiliations:** 1 Department of Pediatric and Preventive Dentistry, Saveetha Dental College and Hospitals, Saveetha Institute of Medical and Technical Sciences, Saveetha University, Chennai, IND; 2 Department of Pediatric and Preventive Dentistry, Saveetha Dental College and hospitals, Saveetha Institute of Medical and Technical Sciences, Saveetha University, Chennai, IND

**Keywords:** kedo nano plus, kedo s plus, kedo s square, pediatric rotary files, pulpectomy, pulptherapy

## Abstract

A 4.5-year-old female child presented to the Department of Pediatric Dentistry with the chief complaints of sharp, localized pain in her lower left and right back teeth persisting for a week, indicative of irreversible pulpitis in teeth 74, 75, and 85. A single-visit pulpectomy was planned for all affected teeth, followed by full coronal restoration in two separate visits. Before the procedure, informed digital consent was taken from the parents. A topical anesthetic agent and inferior alveolar nerve block were administered for effective anesthesia. Rubber dam isolation was performed to ensure aseptic conditions, and access cavity preparation was carried out using appropriate burs. Biomechanical preparation (BMP) was performed using Kedo S Square (Kedo Dental, Chennai, India) in tooth 74, Kedo S Plus (Kedo Dental) in tooth 75, and Kedo Nano Plus (Kedo Dental) in tooth 85, with specific instrumentation techniques as per literature guidelines. The root canals were thoroughly cleaned and shaped to facilitate optimal disinfection and obturation. This case demonstrates the successful management of irreversible pulpitis in the lower primary molar using single-visit pulpectomy with Kedo single filing systems, highlighting the importance of effective BMP in pediatric endodontics.

## Introduction

Long-term dental caries or trauma involving the pulp leads to irreversible pulpal inflammation, abscess formation, or necrosis of the tooth nerve. Diagnosis relies on clinical examination and radiographic findings, such as spontaneous toothache, facial or gum swelling, or evidence of furcal lesion or root resorption on X-rays [1]. The various treatment options include extraction of the tooth followed by suitable space maintainer therapy and lesion sterilization tissue repair is another treatment where pulpal tissue is replaced with suitable antibiotics [2]. A pulpectomy is the most definitive treatment option in necrotic teeth with no evidence of root resorption, where the root canals are disinfected and obturated with a suitable material that helps retain the tooth till exfoliation [3].

Biomechanical preparation (BMP) stands out as a crucial predictive factor in determining the effectiveness of pulpectomy [4,5]. Adequate cleaning and shaping of root canals not only aid in the elimination of infected tissue but also create a pathway for irrigants to penetrate the apical third of the root canals and allow easy flow of obturating material [6]. Kedo-S (Kedo Dental, Chennai, India) is the first-generation rotary file system specifically designed for pediatric endodontics [7,8]. It features modified length, taper, and tip diameter to ensure efficient and comfortable pulpectomy procedures. Over time, the Kedo file system has evolved through six generations, with the latest three being single filing systems, reflecting ongoing advancements in endodontic technology and practice.

Here in the current case report three different single-filing rotary instruments for root canal therapy have been employed on three separate mandibular molars in a single patient, each uniquely designed with distinct metallurgy, cross-sectional designs, and variably variable tapers. The Kedo S Square (P1), Kedo S Plus (P1+), and Kedo Nano Plus (PN+) are three such files specifically created for the preparation of primary root canals in primary molar teeth. These files are effective even when addressing the varied anatomy of primary lower first and second molars. Utilizing different file systems allows the practitioner to leverage the specific strengths of each, potentially improving clinical outcomes like quality of obturation, post-endodontic pain, reducing treatment time, and enhancing patient comfort. This case report aims to highlight the unique advantages and specific applications of each system in clinical practice.

## Case presentation

A 4.5-year-old female child presented to the Department of Pediatric Dentistry with a chief complaint of pain in her lower left and right posterior teeth persisting for the past two weeks. The pain was characterized as sharp, spontaneous, and localized to the posterior teeth on both sides, with nocturnal occurrences. Her past medical history was unremarkable. Clinical examination revealed deep Class I caries in teeth 74, 75, and 85. The radiographic evaluation confirmed the presence of caries involving the enamel and dentin, extending into the pulpal space in these teeth. Based on these findings, a diagnosis of irreversible pulpitis was established for teeth 74, 75, and 85.

The treatment plan involved a single-visit pulpectomy for teeth 74, 75, and 85, followed by full coronal restoration in two separate visits (85 in one visit and 74 and 75 in another). The treatment outcomes and potential complications were thoroughly explained to the parents, and digital informed consent was obtained before the procedure. All treatments were performed by a single-trained pediatric dentist. During the diagnostic visit, the child exhibited positive behavior, as assessed by the Frankl Behavior Rating Scale. In the second visit, the child underwent dental prophylaxis and was introduced to all the dental instruments and materials to be used in the subsequent visit, following a systematic desensitization approach. The child was prescribed syrup AMOX 125 mg/5 mL thrice a day for three days and analgesic syrup IBUCLIN JUNIOR 5 mL to be taken when she experienced pain. She remained on analgesic medication until her third visit, during which the chief complaint was addressed.

During the third visit, a pulpectomy was performed on tooth 85, followed by the placement of a stainless steel crown. In the fourth and final visit, pulpectomies were performed on teeth 74 and 75, and stainless steel crowns were placed on both teeth during that visit.* *The procedural steps for pulpectomy included the application of a topical numbing agent for 3 minutes under suction isolation (Precaine B, Pascal International, Bellevue, WA) and an inferior alveolar nerve block administered using a 2.5 mL syringe with a 25-gauge long needle (UNOLOK single-use syringe, Hindustan Ltd., Chennai, India) containing 2% lignocaine with 1:200,000 adrenaline (LOX* 2% ADRENALINE, Neon Laboratories Limited, India). Isolation was done using a rubber dam (GDC Marketing, Mumbai, India). Caries removal and access opening were performed using a No.4 round diamond bur in a high-speed handpiece. Following the initial access, an Endo Access bur (EA 20, Mani, Inc., Tokyo, Japan) was used to remove the roof of the access cavity. The coronal pulp was removed with a sharp spoon excavator. A no. 15 K-file (Mani, Inc.) was used to check patency in all canals, and the working length was determined using Ingle’s radiographic method. The working lengths of the root canals were measured as follows: for tooth 74, the mesiobuccal (MB) and mesiolingual (ML) canals measured 13.5 mm, while the distobuccal (DB) and distolingual (DL) canals measured 13 mm. For tooth 75, the MB and ML canals measured 14 mm, and the DB and DL canals measured 13.5 mm. For tooth 85, the MB canal measured 14.5 mm, the ML canal measured 14 mm, and the DB and DL canals each measured 13.5 mm. BMP was done to determine the working length using different single filing systems in each tooth, which is mentioned in Table 1.

**Table 1:** Biomechanical preparation (BMP) using Kedo files.

Kedo S Square (P1) in 74	Kedo S Plus (P1+) in 75	Kedo Nano Plus (PN+) in 85
Initial preparation by size 15 K-file in all the three canals, i.e., MB, ML, DB, and DL, followed by Kedo-S Square (P1) (Kedo Dental, Chennai, India) in all the three canals in lateral brushing motion [9].	Initial preparation by size 15 K-file in all the four canals, i.e., MB, ML, DB, and DL canals, followed by Kedo-S Plus (P1+) (Kedo Dental) in all the four canals in lateral brushing motion [10].	Initial preparation by size 15 K-file in all the four canals, i.e., MB, ML, DB, and DL canals, followed by Kedo Nanoplus (PN+) (Kedo Dental) in all the four canals in lateral brushing motion.

**Figure 1:**
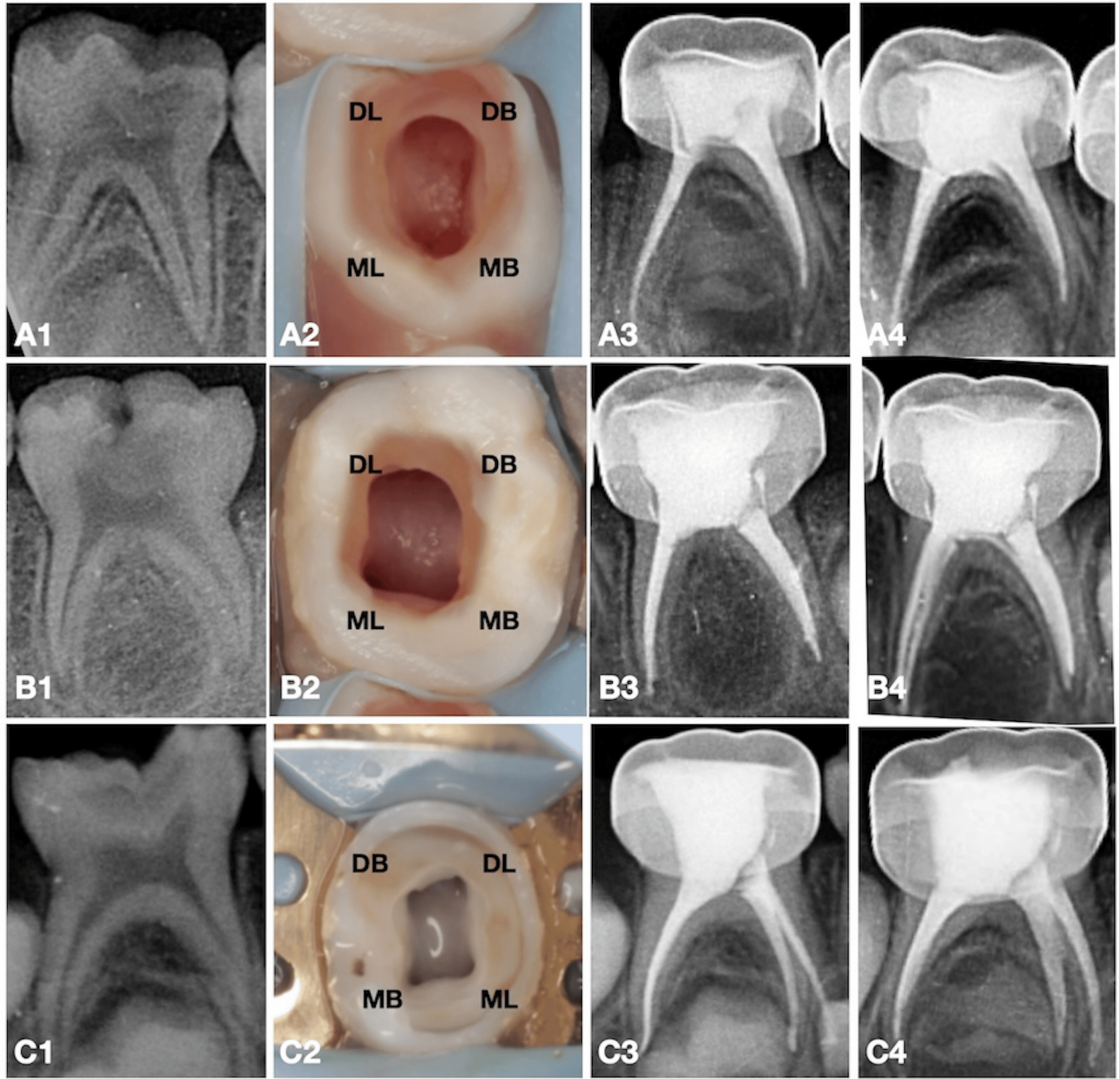
Preoperative radiographs, canal post-preparation, and postoperative radiographs of the case. A1, A2, A3, A4 represent the preoperative radiograph, post-canal preparation, immediate postoperative radiograph, and six-month follow-up radiograph of teeth 74 treated with Kedo S Square; B1, B2, B3, B4 represent the preoperative radiograph, post-canal preparation, immediate postoperative radiograph, and six-month follow-up radiograph of teeth 75 treated with Kedo S Plus; C1, C2, C3, C4 represent the preoperative radiograph, post-canal preparation, immediate postoperative radiograph, and six-month follow-up radiograph  postoperative radiograph of teeth 85 treated with Kedo Nano Plus. MB, mesiobuccal canal; ML, mesiolingual canal; DB, distobuccal canal; DL, distolingual canal

Rotary files were used with an X-Smart Plus endodontic motor (Dentsply Maillefer, OK) operating at 300 rpm and 2.5 N/cm torque until the determined working length was achieved. A 17% EDTA solution (Endo Prep RC) was utilized for lubrication during instrumentation, with saline (Fresenius Kabi India Pvt. Ltd., Mumbai, India) used for final irrigation. Approximately 10 mL of saline was used for irrigation for each tooth. After drying the canals with appropriate paper points, obturation was performed using a calcium hydroxide-iodoform paste with a pressure syringe technique (Metapex, Meta Biomed Co., Ltd., Korea). Subsequently, the access cavities were filled with type II glass ionomer cement (GC, Mumbai, India) to provide a coronal seal. During the same appointment, the teeth were restored with preformed stainless steel crowns (3M ESPE, Seefeld, Germany). The patient was reviewed after six months. During the recall visit, the patient remained asymptomatic with no clinical or radiographic signs of reinfection or resorption of obturating material. Preoperative radiographs, clinical images post-BMP, immediate postoperative radiographs, and six-month follow-up radiographs of all three teeth are shown in Figure 1.

## Discussion

Manual root canal instruments were typically used for cleaning and shaping the primary root canals in pulpectomy procedures. Barr was the first to use rotary files designed for permanent teeth in these procedures [11]. However, using both hand files and rotary files intended for permanent teeth often led to procedural errors such as ledging, zipping, canal transportation, and apical blockage, as these tools were not suited to the anatomy of primary root canals [12]. Now, pediatric-specific rotary files have been introduced, specifically designed for the tortuous nature of the canals of the primary teeth. These specialized rotary files have undergone numerous improvements over generations, enhancing their compatibility with primary root canal anatomy.

Kedo-S, introduced in 2017, was the world's first pediatric rotary file system [13]. Over the years, the Kedo files have undergone numerous changes, adapting metallurgies, cross-sections, and diameters. The current trend is to use a single filing system, including the fourth-generation Kedo-S Square, the fifth-generation Kedo-S Plus, and the latest sixth-generation Kedo-Nano Plus [14]-the specification of these file systems mentioned in Table 2.

**Table 2:** Specifications of the files. Data Source: www.kedofiles.com. Permission and attribution for the use of images in this manuscript were obtained from the owner of the website and the product with reference number 20240503/0849. CM, controlled Memory; NiTi, nickel titanium; VV, variably variable; rpm, revolution per minute; Tq, torque

	Kedo S Square	Kedo S Plus	Kedo Nano Plus
Length	File length - 16 mm	File length - 16 mm	File length - 16 mm
	Flute length - 12 mm	Flute length - 12 mm	Flute length - 12 mm
Metallurgy	CM wire technology	CM wire technology	CM wire technology
	NiTi heat-treated files with titanium oxide coating	Dual metallurgy	NiTi heat-treated files with nanoparticle coated
		Coronal portion - heat treated	
		Apical portion - heat treated with titanium oxide coating	
Cross-section	Dual	Triangular	Triangular
	Apical 5 mm - triangular		
	Coronal region - teardrop		
Taper	VV taper - 4% to 8%	VV taper - 4% to 8%	VV taper - 4% to 8%
rpm	250-300	250-300	250-300
Tq	2.2-2.4	2.2-2.4	2.2-2.4

**Figure 2:**
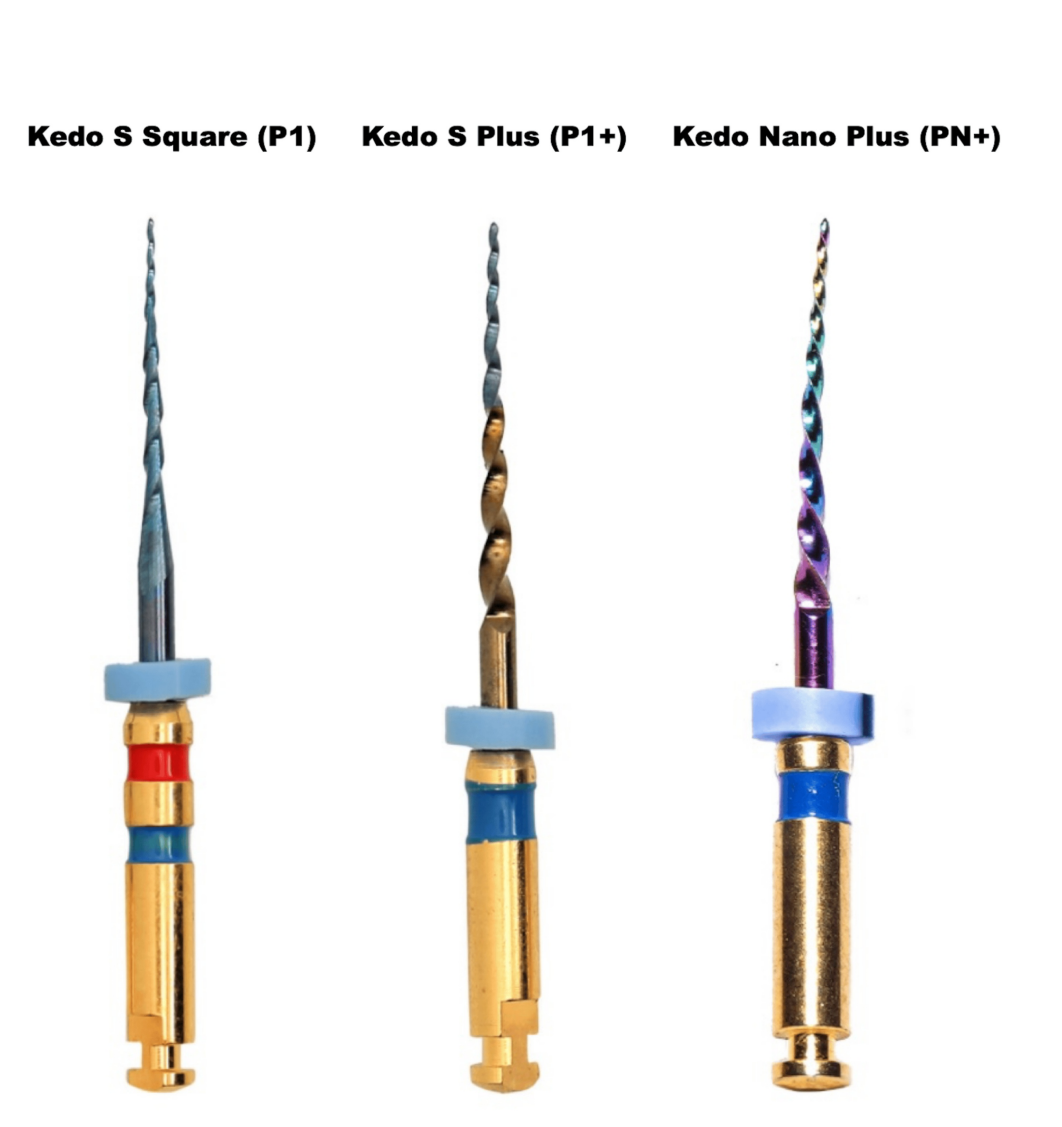
File systems used in this case. Image source: www.kedofiles.com. Permission and attribution for the use of images in this manuscript were obtained from the owner of the website and the product with reference number 20240503/0849.

Picture of three distinct file systems used in this cases have been shown in Figure 2.

These advancements help pediatric dentists reduce chairside time while providing effective shaping abilities. In this case report, we utilized all these single filing systems for a pulpectomy procedure in a mandibular primary molar.

The fourth-generation file, Kedo-S Square, was used by several researchers who compared and identified its abilities. They found that postoperative pain was significantly reduced when using Kedo-S Square compared to sequential filing systems, and the quality of obturation was optimal. Lakshmanan et al. also noted the file's fracture incidence, mentioning that it can be used for up to 12 molar teeth pulpectomies [15,16,17]. On visually analyzing the postoperative radiograph of tooth 74, we could appreciate thinner obturation along the entire length of the canals, which depicts minimal canal volumetric changes after the chemomechanical preparation of the canals as compared to preoperative radiograph. 

The fifth-generation file, Kedo-S Plus, has been evaluated by researchers for its ability to canal centering, the volumetric change after cleaning and shaping, and the apical extrusion of debris (a major factor in postoperative pain). The Kedo-S Plus file showed a significant reduction in the extrusion of apical debris [10]. It also maintained the centering ability throughout the canal and showed moderate volumetric changes [18]. Similarly, in our case report, the file effectively removed the pulp and shaped the canal optimally, allowing for proper obturation. On visually analyzing the postoperative radiograph of tooth 75, we observed a wider coronal preparation with minimal apical preparation of the canals which is more significantly noticed in distal canals. This aggressive coronal preparation could be due to the dual metallurgy of the file system used (Kedo-S Plus) [19]. Heat-treated coronal third had an aggressive canal preparation, which had resulted in wider coral preparation of the canals, whereas a titanium oxide-coated apical third of the file provided a minimal canal preparation due to increased flexibility. 

The latest generation file, Kedo-Nano Plus, is coated with nanoparticles along its length, making it highly flexible and capable of withstanding torsional forces in curved canals. As mentioned, we were able to clean the apical portion optimally, which is crucial due to the ramifications in this area, and were able to provide optimal obturation. On visually analyzing the postoperative radiograph of tooth 85, we observed a minimal apical preparation of the canals but slightly wider coronal preparation but not similar to Kedo-S Plus, which is significantly noticed in distal canals. Although having similar metallurgy and dimensions of Kedo-S Plus, Kedo Nano Plus had an additional nano coating [20]. This nano coating of the file was added to reduce the file fracture, which could have reduced the aggressive nature of the file in the coronal aspect, thereby leading to slightly reduced preparation in the coronal third of the canals.

The clinical significance of this case report highlights the importance of selecting the appropriate filing system based on specific clinical requirements. For clinicians aiming for minimal canal preparation and resulting in thinner obturation, Kedo S Square is the recommended file system. For cases requiring minimal apical preparation with a slightly wider coronal preparation to facilitate the flow of obturating materials, Kedo S Plus is the preferred choice. Additionally, for clinicians who seek similar benefits to Kedo S Plus but with enhanced file longevity, Kedo Nano Plus is advisable. These options allow for tailored clinical approaches, ensuring optimal patient outcomes.

## Conclusions

This case report demonstrates the effective management of irreversible pulpitis in a 4.5-year-old child through single-visit pulpectomy and subsequent coronal restorations using stainless steel crowns. The treatment was meticulously executed, employing systematic desensitization techniques to ensure the child's comfort and cooperation throughout the procedures. The study further evaluated the efficacy of three distinct single-file pediatric rotary systems in performing pulpectomy on lower molars in a pediatric patient. Each rotary system exhibited unique features tailored to pediatric endodontics, providing advantages such as enhanced safety, efficiency, and patient comfort. The use of Kedo S Square, Kedo S Plus, and Kedo Nano Plus rotary files facilitated precise BMP, contributing to the success of the endodontic therapy. By incorporating these advanced rotary systems into our clinical practice, we can improve the safety and effectiveness of pediatric endodontic procedures. This case highlights the potential for these technologies to enhance treatment outcomes, advancing the field of pediatric dentistry and ensuring optimal oral health for the pediatric population.
